# Immunological mechanisms in steroid-induced osteonecrosis of the femoral head

**DOI:** 10.3389/fimmu.2025.1626617

**Published:** 2025-08-13

**Authors:** Zhilei Yin, Longfei Wu, Jiwei Huang, Haiyan Zhao

**Affiliations:** ^1^ The First Clinical College of Medicine, Lanzhou University, Lanzhou, China; ^2^ Department of Orthopedic, The First Hospital of Lanzhou University Gansu, Lanzhou, China

**Keywords:** glucocorticoid, immune cells, osteoimmunity, macrophage polarization, SONFH

## Abstract

Steroid-induced osteonecrosis of the femoral head (SONFH) represents a prevalent and complex orthopedic condition, intricately linked to multifaceted dysregulation of the immune system. Prolonged administration of high doses of GCs (GCs) represents a major cause of non-traumatic osteonecrosis of the femoral head (ONFH), with its pathogenesis rooted in the interaction between immune cell dysfunction and imbalances in bone metabolism. This review systematically explores the molecular mechanisms through which GCs induce osteonecrosis via immunological pathways, with emphasis on the impact of macrophage polarization imbalance on the disruption of the bone immune microenvironment. This encompasses the metabolic reprogramming of macrophages and the involvement of critical signaling pathways. This study sought to establish a comprehensive theoretical framework for the immunological regulatory mechanisms underlying SONFH, to provide a detailed understanding of the mechanisms by which GCs induce bone immune disorders, and to offer a robust theoretical foundation for the formulation of early intervention strategies.

## 1 Introduction

Osteonecrosis of the femoral head (ONFH) is an orthopedic disease caused by insufficient blood supply to the femoral head, leading to bone tissue necrosis and incomplete repair, ultimately causing joint destruction and functional impairment. Early diagnosis of osteonecrosis of the femoral head is particularly challenging, and the disease is associated with high disability rates (1). Osteonecrosis of the femoral head can be divided into traumatic and non-traumatic depending on the underlying cause. Traumatic osteonecrosis of the femoral head typically occurs following hip trauma and is a common cause of femoral head necrosis (2, 3). Non-traumatic osteonecrosis of the femoral head is more common in middle-aged and young people aged 20-50, with hip joint involvement in about 80% of cases. It is mainly caused by long-term use of GCs, long-term excessive alcohol consumption (4), decompression sickness, autoimmune diseases, hemoglobinopathies, and certain metabolic diseases (hyperlipidemia, diabetes, etc.). In addition, obesity, smoking, radiotherapy, and pregnancy reportedly increase the risk of ONFH (5). ONFH, as one of the most common refractory diseases in orthopedics, exhibits an annual incidence rate of about 1.5-3.0 per 100,000 people across different countries (5). Currently, although some countries have screened their populations for osteonecrosis, there is still no global epidemiological report on ONFH. China has the largest number of ONFH patients, with a large-scale epidemiological survey of non-traumatic ONFH in 2015 estimating that there are 8.12 million cases of ONFH in the Chinese population aged 15 and above (6), with 38.27% of patients having a history of glucocorticoid use.

Since the seminal investigations into immune cell-derived osteoclast-activating factors dating back to 1972, it has become increasingly evident that the skeleton, functioning as a highly dynamic and metabolically active organ, is significantly modulated and profoundly impacted by the intricate interplay with the immune system (7). It has been established that osteocytes and immune cells not only coexist within the marrow cavity but also share a plethora of regulatory molecules. In the processes of bone necrosis and bone remodeling, signaling from a diverse array of immune cells plays a pivotal role. Therefore, this review focuses on elucidating the central role of macrophage polarization imbalance in SONFH and its regulatory mechanisms, encompassing metabolic reprogramming and critical signaling pathways. Subsequently, it briefly examines the contributions of other immune cells—including T cells, B cells, neutrophils, dendritic cells, and mast cells—to disease progression and their interplay with macrophages (8). Analogous to the established mechanism that smoking can induce skeletal destruction via its interaction with the immune system (9), a comprehensive understanding of the immune mechanisms underlying hormone-induced osteonecrosis of the femoral head warrants a single-cell perspective. Indeed, a comprehensive examination of the multifaceted influences exerted by a diverse array of immune cells on the pathogenesis of this condition is essential (10).

Although significant progress has been made in osteoimmunology toward understanding SONFH, the precise mechanisms through which GCs remodel the bone-immune microenvironment, leading to immune cell dysfunction (particularly in macrophages), thereby disrupting bone metabolic homeostasis (including angiogenesis, osteoblast/osteoclast balance, and adipogenic transdifferentiation) and ultimately triggering irreversible osteonecrosis remain incompletely elucidated. Therefore, we systematically dissect the molecular mechanisms by which GCs induce ONFH via immunological pathways, with a particular focus on how immune cells (especially macrophage polarization imbalance) and their mediated signaling networks disrupt the bone-immune microenvironment. This article aims to provide references and insights for exploring the pathogenesis of this condition and developing early treatment strategies.

## 2 Macrophage polarization influences the progression of SONFH

Within the bone-immune microenvironment, macrophages can modulate bone metabolism by releasing various cytokines and exosomes (11). During the initial inflammatory phase, a specific macrophage subtype preferentially accumulates at inflammatory foci, essential for the elimination of extracellular noxious agents and the clearance of metabolic byproducts generated within the body. Conversely, during the advanced stages of inflammation, a distinct macrophage subset becomes more prominent, essential for the suppression of inflammatory responses and the promotion of tissue repair. Throughout the inflammatory process, the relative proportion of these two macrophage populations fluctuates over time, and the phenotypic states are capable of interconversion. Accordingly, macrophages are categorized into two distinct functional phenotypes: pro-inflammatory (M1) and anti-inflammatory (M2). Macrophages are typically categorized into two distinct types: pro-inflammatory (M1) and anti-inflammatory (M2) (12, 13). M1 macrophages classically exhibit pro-inflammatory properties, and can be elicited by lipopolysaccharide (LPS) or Th1 cytokines, such as IFN-γ and TNF-α (14). M1 macrophages typically produce IL-12, COX-2, iNOS, PGE2, as well as TNF-α, which serve as the cornerstone in the defense against infections. Conversely, M2 macrophages exhibit pro-healing and anti-inflammatory properties, producing cytokines such as IL-10, IL-4, CCL1, and CCL18 (15). M2 macrophages secrete IL-10, which has a direct pro-osteogenic effect (16), with these molecules are potentially implicated in the process of tissue regeneration.

In animal models of ONFH, the increased abundance of M1 macrophages stimulated by necrotic bone elicits a pro-inflammatory response. Conversely, M2 macrophages are pivotal in the resolution of inflammation and the reparative regeneration of compromised tissues, especially during the advanced stages of ONFH pathogenesis. The phenotypic shift from M1 to M2 macrophages can significantly enhance osteoblast survival, mitigate inflammatory cytokine production, and ameliorate the clinical manifestations of ONFH (17). Research has substantiated the perspective that M2 macrophages can facilitate osteoblast differentiation (18).

Research has demonstrated a notable increase in M1 macrophages within the femoral head and bone marrow blood samples obtained from patients afflicted with SONFH (19). Furthermore, it has been observed that M1 macrophages exert a significant inhibitory effect on the osteogenic differentiation of BMSCs, which is mediated through the extensive downregulation of genes associated with osseous mineralization (20). Nevertheless, there is compelling evidence that M1 macrophages may exert beneficial effects, particularly during the initial phase of bone repair. Gema Vallés and colleagues revealed that cytokines secreted by M1-polarized macrophages enhanced the adhesive and migratory properties of MSCs. Florence Loi and colleagues revealed that all macrophage subtypes could augment the osteogenic potential of MC3T3 cells (21). While IL-10, secreted by M2 macrophages, exerts a more robust effect, it augments the osteogenic differentiation of BMSCs and modulates the expression of osteogenic markers, indicative of its pivotal role in bone formation processes (22).

These studies overlap in their assertion that M1 and M2 macrophages play distinct roles in bone regeneration. Nevertheless, an imbalance in M1/M2 polarization markedly influences the process of bone regeneration and the progression of SONFH.

### 2.1 Metabolic reprogramming can influence macrophage polarization

**Figure 1:**
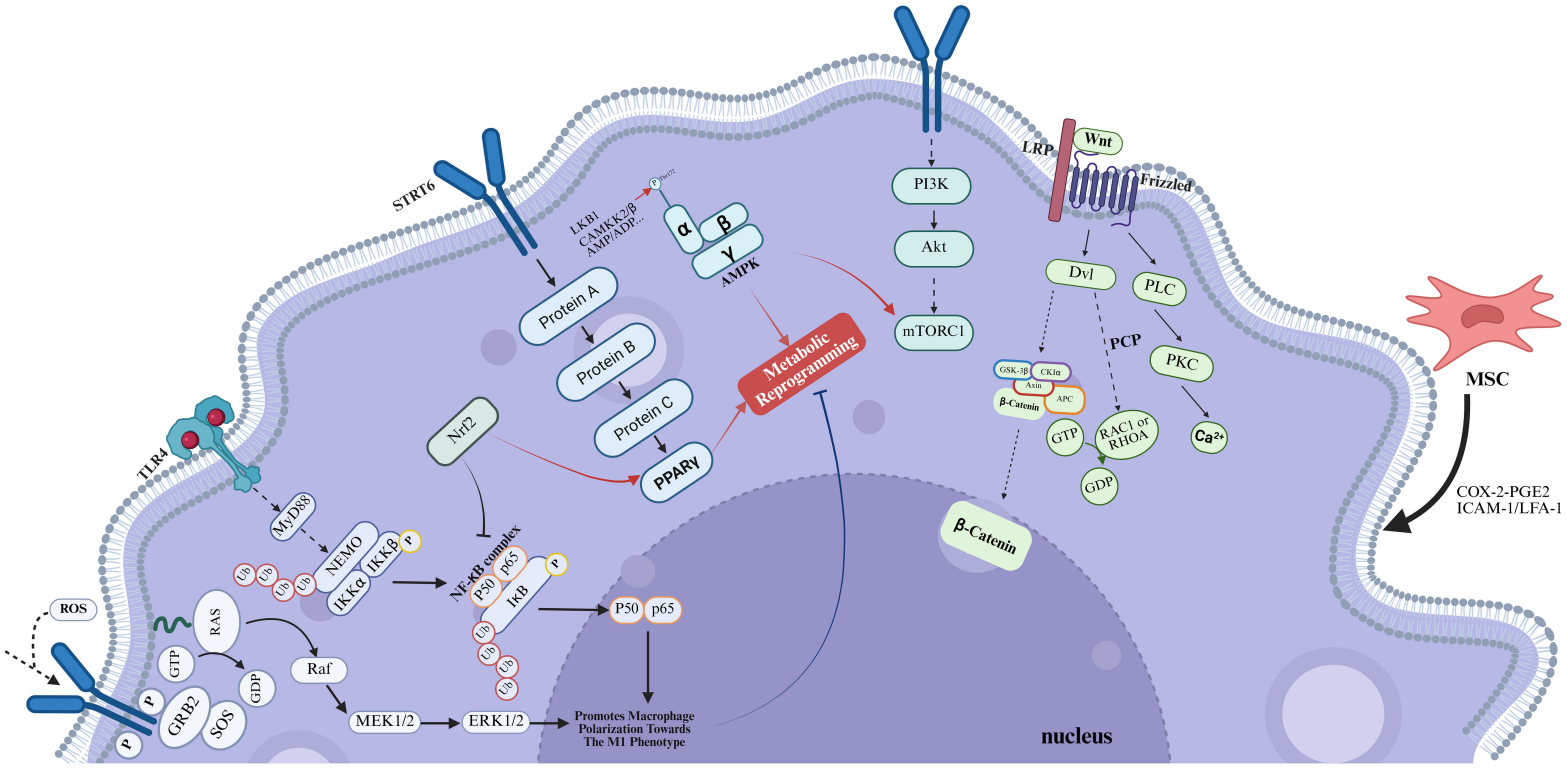
In the context of SONFH, chronic administration of high-dose GCs results in a significant imbalance in macrophage polarization. Figure created using BioRender.

The polarization of M1/M2 macrophages is intrinsically linked to metabolic reprogramming, where the enhancement of glycolysis and the impairment of mitochondrial oxidative phosphorylation play significant roles (23, 24). In mammals, there are two important subfamilies of MAPKs—extracellular signal-regulated kinases 1 and 2 (ERK1/2), c-Jun amino-terminal kinases 1 to 3 (JNK1 to 3)—that coordinately regulate cell proliferation, differentiation, motility, and survival (25). In macrophages, the MAPK/ERK and MAPK/JNK pathways can regulate the expression of mitochondrial respiratory chain complexes, which in turn modulate basal respiration, ATP production during OXPHOS, and glycolysis in BMDMs, ultimately affecting the M1/M2 polarization (**Figure 1**). The research findings demonstrate that inhibition of the MAPK signaling pathway effectively suppresses the polarization of M1 macrophages while concurrently promoting the polarization of M2 macrophages (26, 27). This phenomenon is consistent with the observation that GCs increase the phosphorylation levels of molecules within the MAPK pathway and induce an imbalance in macrophage polarization (28). This process plays a crucial role in immune regulation and the repair following bone injury. However, in the aforementioned process, GCs may be intricately linked to the involvement of ROS. For instance, prolonged high-dose administration of GCs can lead to excessive activation of ERK, which may subsequently induce ROS and apoptotic signaling. This cascade of events can disrupt the M2 polarization capacity of macrophages (29). Moreover, ROS induced by GCs may activate the ASK1-JNK signaling pathway, thereby promoting the polarization of macrophages towards the M1 phenotype (30). In summary, GCs can inhibit the polarization of macrophages towards the M2 phenotype via the MAPK signaling pathway, thereby promoting the occurrence of SONFH.

LPS and IFN-γ-activated M1 macrophages are associated with a metabolic shift from oxidative phosphorylation (OXPHOS) to glycolysis, which not only sustains the viability and pro-inflammatory activity of M1 macrophages but also, by inhibiting OXPHOS, reduces the reliance on OXPHOS, thereby inhibiting the polarization and expression of genes associated with M2 macrophages, such as Arg-1 and Mrc1 (31). Notwithstanding the anti-inflammatory actions of GCs, their modulation of mitochondrial function exhibits a paradoxical profile. The seminal work by Ulrich Stifel et al. revealed that GCs could intricately regulate the production of glutathione and ROS, as well as the functionality of the tricarboxylic acid cycle (TCA cycle), via a HIF1α-dependent mechanism, ultimately inhibiting glycolysis (32). This observation seemingly contradicts the prevailing notion that GCs disrupt the M1/M2 macrophage polarization balance. However, this finding underscores the necessity to meticulously consider the dosage and duration of GCs exposure when evaluating their detrimental effects within the context of disease progression. In this context, research by Gee Euhn Choi et al. provided further insights, demonstrating that prolonged exposure to high-dose GCs is associated with increased mitochondrial ROS production and a concomitant reduction in mitochondrial membrane potential (33). These findings not only corroborate the widely accepted hypothesis regarding glucocorticoid-induced mitochondrial dysfunction but also align with the preconditions necessary for the pathogenesis of SONFH.

Enhanced glycolysis in M1 macrophages generates lactate and ROS that not only affect macrophage autocrine signaling but also diffuse spatially to inflict damage on neighboring bone cells, including osteocytes, osteoblasts, and endothelial cells, inducing apoptosis or necrosis. Concurrently, GCs-induced mitochondrial dysfunction, characterized by elevated ROS production and diminished membrane potential, constitutes a critical mechanistic link connecting immunometabolic dysregulation with osteocyte death.

### 2.2 GCs promote the NF-κB signaling pathway to facilitate M1 macrophage polarization

During the progression of SONFH, M1 macrophages demonstrate increasing abundance. Following the timely ablation of M1 macrophages, a reduction in NF-κB expression and the suppression of apoptosis were observed, culminating in a markedly decelerated progression of SONFH (34). It is well-established that NF-κB-related signaling pathways are key routes associated with the activation of M1 induced by TLR ligands and IFN-γ. Besides, a multitude of therapeutic approaches have been identified to ameliorate SONFH by targeting the NF-κB signaling pathway (35, 36). The TLR4/NF-κB signaling cascade is a quintessential pathway that drives macrophage polarization. Activation of the NF-κB pathway occurs upon the interaction of TLR4 with LPS on the macrophage surface, a process that is reliant on either MyD88 or IRF3, thus promoting the M1 macrophage polarization (**Figure 1**) (37). During the pathogenesis of SONFH, damaged vascular endothelial cells and necrotic bone tissue can trigger the activation of this signaling pathway. This activation facilitates the dimerization of Pyruvate Kinase M2 (PKM2), thereby augmenting the synthesis of HIF-1. Consequently, this cascade of events induces a metabolic reprogramming of macrophages, shifting their phenotype towards the pro-inflammatory M1 subtype (38). Apart from TLR4, the upstream regulators of NF-κB encompass SIRT3, ROS, and other related factors, which also play a role in the differentiation of osteoclasts and osteoblasts (39, 40), suggesting that GCs may exert their effects on the NF-κB signaling pathway via multiple, potentially distinct pathways, thereby modulating the equilibrium of M1/M2 macrophage polarization.

The NLRP3 inflammasome, a tripartite complex comprising NLRP3, caspase-1, and ASC (apoptosis-associated speck-like protein containing a CARD), modulates M1 polarization (41) by orchestrating the activation of caspase-1 and the subsequent release of inflammatory cytokines IL-1β and IL-18. The activation of inflammasome signaling cascades results in heightened local inflammatory responses and enhanced osteoclastogenesis, consequently precipitating bone resorption and destruction (42). The NF-κB signaling pathway is involved in its initiation steps, and its general activation pathway requires the assistance of pattern recognition receptors (PRR) (43). PRRs are located on the surface of innate immune cells and can directly recognize pathogen-associated molecular patterns (PAMPs) or specific molecular structures during host cell apoptosis, playing a role in immune recognition and response. These receptors include Toll-like receptors (TLRs), C-type lectin receptors (CLRs), scavenger receptors, retinoic acid-inducible gene 1 (RIG1)-like helicase receptors (RLRs), and NOD-like receptors (NLRs) (44). Following the binding of stimulatory factors such as LPS to TLR4, NF-κB is activated, leading to an increase in NLRP3 expression, which in turn promotes M1 polarization. While this appears to be a straightforward mechanism, the TLR4/NF-κB signaling pathway does not function alone. Firstly, TLR4 can simultaneously activate both the MAPK and NF-κB signaling pathways (45). Secondly, the MAPK signaling pathway can enhance the transcriptional activity of NF-κB through phosphorylation, thereby promoting the expression of pro-inflammatory cytokines. Concurrently, NF-κB can modulate the activity of MAPK by regulating the expression of certain kinases within the MAPK signaling cascade (46). Peroxisome proliferator-activated receptors (PPARs), which are part of the nuclear receptor superfamily, function as ligand-activated transcription factors. Their pivotal role in macrophage polarization is characterized by their capacity to suppress the initiation of the pro-inflammatory M1 signaling cascade and concurrently enhance the expression of anti-inflammatory M2 signaling pathways (**Figure 1**) (47). Existing literature has substantiated the existence of an interplay between the PPARγ and NF-κB signaling cascades. The activation of PPARγ is known to exert an inhibitory effect on the NF-κB pathway through a direct interaction with the p65 subunit of NF-κB, culminating in the attenuation of signal transduction (48), and exert an antagonistic effect on NLRP3 (49). Beyond this, Nrf2 can activate the PPARγ pathway and inhibit NF-κB signaling (50); increased AMP-activated protein kinase (AMPK) activity can block LPS-induced phosphorylation of NF-κB p65 (51). Besides, other pathways such as the PI3K/AKT/mTORC1 pathway can also interact with the NF-κB pathway, thereby affecting macrophage polarization and osteoclast differentiation, among other processes (52, 53). This indicates the presence of complex interplay mechanisms among the signaling pathways that influence macrophage polarization.

GCs can directly or indirectly influence bone cells by modulating NF-κB signaling in immune cells. Concurrently, NF-κB activation within osteoblasts and osteoclasts directly regulates bone metabolic genes. Following NF-κB activation, pro-inflammatory cytokines such as TNF-α and IL-1β secreted by M1 macrophages further suppress osteoblast differentiation/function while activating osteoclastogenesis. Consequently, NF-κB constitutes a pivotal nexus linking innate immunity (macrophages) to bone resorption, a process subject to regulation by additional signaling pathways.

### 2.3 The PPARγ signaling pathway promotes macrophage polarization towards the M2 phenotype

PPARγ is a pivotal regulator in adipogenesis. Adipogenic stimuli facilitate the terminal differentiation of preadipocytes into mature adipocytes through the epigenetic activation of PPARγ. The coordinated maintenance of adipocyte gene expression is orchestrated by the interplay between PPARγ and CCAAT/enhancer-binding proteins (C/EBP) transcription factors (54). GCs exert a profound influence on the osteogenic-adipogenic differentiation equilibrium of MSCs through a multitude of signaling cascades. These pathways culminate in the modulation of transcription factors, including PPARγ and C/EBPs, thereby directing MSCs towards adipogenic differentiation (55). GCs-induced M1 macrophages may also enhance the adipogenic differentiation of BMSCs by secreting exosomes that deliver miR-1a-3p to BMSCs, thereby targeting and suppressing the expression of C/EBPZ (20). Nonetheless, this observation appears to be inconsistent with the previously discussed notion that PPARγ facilitates the expansion of the M2 macrophage phenotype. It is well established that GCs can induce PPARγ expression, thereby augmenting its role in adipocyte differentiation and metabolic regulation. Given this context, it raises the question of how GCs, which are known to promote the polarization of M1 macrophages, could potentially enhance the M2 macrophage population through PPARγ. Research conducted by Michael Heming suggested that the primary effect of GCs on the inflammatory cytokine response is predominantly PPARγ-independent (56).

As a pivotal receptor involved in modulating the inflammatory and anti-inflammatory differentiation pathways of macrophages, PPARγ is not solely engaged in crosstalk with NF-κB. Regarding PPARγ, its upstream pathways include at least the STAT and SIRT pathways, and through pSTAT6, Ilaha Isali et al. revealed the crosstalk between PPARγ and Nrf2 (57, 58). The Nrf2 signaling pathway is also involved in the pathogenesis of SONFH and plays a pivotal role in the polarization of macrophages (59). In the MAPK signaling pathway, MEK, which is located between RAS and the previously mentioned EAK, is a key regulatory factor in the induction of PPARγ that promotes the M2-type polarization. He Lizhi et al. consistently revealed the intricate interplay between PPARγ and energy metabolism (60). We previously mentioned the capacity of mTORC1 to enhance NF-kB phosphorylation, thereby impacting macrophage polarization. However, the key mediator in this mechanism is mTOR. The mTOR signaling transduction cascade encompasses two distinct multimeric complexes: mTOR Complex 1 and mTOR Complex 2 (mTORC1/2) (61). The mTORC1/HIF-1α signaling axis, in conjunction with augmented glycolytic activity, can facilitate the M1 polarization of macrophages. Furthermore, the mTORC2/PPAR-γ signaling cascade can orchestrate fatty acid oxidation (FAO) to promote M2 polarization. This research not only revealed the divergent roles of mTOR in the context of macrophage polarization and linked them with PPAR-γ, but also reestablished a link between macrophage polarization and metabolic reprogramming (62), consistent with the previous findings (63).

Although the primary effect of GCs-mediated modulation of inflammatory cytokine responses may be PPAR-γ-independent, PPAR-γ nevertheless serves as a master adipogenic transcription factor. GC-induced PPAR-γ upregulation constitutes the core mechanism driving BMSCs toward adipogenic rather than osteogenic differentiation. This process represents a critical pathological basis for osteonecrosis. Synergizing with the influence of M2 macrophage phenotypes on BMSCs differentiation—exemplified by mechanisms such as miR-1a-3p delivery—PPAR-γ dysregulation collectively culminates in the terminal outcome of bone necrosis.

### 2.4 GCs suppress AMPK signaling to impede promotes macrophage polarization towards the M2 phenotype

The activation of the AMP-activated protein kinase (AMPK) signaling pathway robustly enhances fatty acid oxidation metabolism, attenuates the production of pro-inflammatory cytokines, and concomitantly augments the secretion of anti-inflammatory cytokines. This metabolic reprogramming is essential for orchestrating the phenotypic switch from pro-inflammatory (M1) to anti-inflammatory (M2) macrophages (**Figure 1**) (64). Wenbin Ye et al. employed the transfection of AMPK-α siRNA into the murine macrophage-like Raw264.7 cell line, resulting in a marked promotion of the M2 phenotypic transition induced by irisin, aligning with the findings of prior research (65). The downstream signaling molecules mainly involve mTOR, NF-κB, PPAR, and Nrf2. AMPKα1 is instrumental in the activation of IL-10 during macrophage polarization orchestrated by the PI3K/AKT/mTORC1 and STAT3 signaling pathways (66), reflecting the intricate interplay between the AMPK and PI3K/AKT pathways.

Notably, AMPK activation is predominantly associated with M2c macrophages, a phenotype we previously highlighted as being inducible by GCs. This association suggests that GCs may effectively orchestrate macrophage polarization toward a reparative phenotype and facilitate inflammation resolution via AMPKα1 (67). This raises the question of whether GCs can polarize macrophages into the M2c phenotype through AMPKα1, thereby ameliorating ONFH. The answer is negative. Although AMPK-α can indeed induce M2 macrophage polarization (68). However, it should be acknowledged that the effects of GCs on AMPK are highly context-dependent, varying across different tissues and durations of exposure. AMPK can enhance osteoblast differentiation, mitigates osteoblast senescence, and suppresses osteoclastogenesis (69, 70). In the necrotic regions of the femoral head in SONFH, AMPK activity is markedly suppressed, resulting in oxidative damage and necroptosis of BMSCs and ultimately contributing to femoral head necrosis (71, 72). This observation is consistent with our prior conclusions. The polarization of M2c macrophages induced by GCs exhibits no significant correlation with AMPK signaling. In summary, the influence of GCs on AMPK in the pathogenesis of SONFH is multifaceted. Although this complexity does not alter the ultimate outcome of SONFH, it highlights the necessity for a more nuanced and comprehensive evaluation of the interplay between GCs and AMPK when investigating related issues.

### 2.5 GCs suppress PI3K/AKT signaling pathway to impede macrophage polarization towards the M2 phenotype

The PI3K/AKT signaling can promote M2-like activation by upregulating the expression of PGC1α, which in turn facilitates mitochondrial biogenesis and OXPHOS. Besides, this pathway promotes the osteogenic differentiation of BMSCs (**Figure 1**) (73). In the investigation of the interplay between GCs and the PI3K/AKT signaling pathway, our research elucidated that GCs exerted a suppressive effect on the PI3K/AKT cascade, which is instrumental in the pathogenesis of SONFH. However, within the realm of ONFH, most studies examining the inhibitory actions of GCs on the PI3K/AKT pathway have focused on their capacity to enhance osteoclast autophagy, thereby promoting osteoclast differentiation, as well as their detrimental effects on BMECs (74–76). Conversely, the influence of GCs on macrophage polarization equilibrium, mediated through the PI3K/AKT pathway, remains largely underexplored. Given the pivotal role of the PI3K/AKT pathway in the progression of SONFH, its potential impact on macrophage polarization represents a critical and under-investigated area that warrants further in-depth exploration. The PI3K/AKT pathway can classically activate mTOR, leading to a polarization bias towards M2 macrophages, which is conducive to the differentiation and maturation of osteoblasts (77). Serum- and glucocorticoid-induced kinase 1 (SGK1) serves as a pivotal Akt-independent mediator within the PI3K/mTOR signaling cascade. Its functionality is intricately linked to the phosphorylation status of mTOR, exerting a significant influence on the differentiation of T helper 1 (Th1) and T helper 2 (Th2) cells, which exists at least in the tumor microenvironment (78). Recent studies have demonstrated that activation of the PI3Kγ/SGK1 signaling pathway can induce the polarization of macrophages towards the M2 phenotype, redirecting research focus towards cardiac fibrosis and atrial fibrillation. Given the pivotal role of SGK1 in modulating cellular responses, research on SGK1 in the field of osteonecrosis holds huge promise (79). A novel nTPG/PLGA/PCL fibrous membrane exhibited responsiveness to both the PI3K/Akt pathway activator 740Y-P and the NF-κB pathway activator PMA in experimental settings. While a direct interaction between the PI3K/AKT and NF-κB signaling pathways was not established, this study provided valuable insights into the potential crosstalk between these two critical pathways. Furthermore, it substantiated the association between macrophage polarization and osteogenic effects (80).

GCs inhibit this signaling pathway, thereby not only impacting macrophage polarization but also directly impairing osteogenesis in BMSCs and compromising the functionality of BMECs.

### 2.6 GCs suppress Wnt signaling pathway to impede macrophage polarization towards the M2 phenotype

During the processes of tissue injury and repair, specific Wnt ligands secreted by macrophages can exert significant regulatory functions. Based on the properties of these ligands and the downstream signaling events they initiate, the Wnt signaling pathway is classified into two major categories: the canonical Wnt pathway and the non-canonical Wnt pathway (81). The canonical Wnt-β-catenin pathway is indispensable for maintaining normal bone homeostasis. This pathway promotes the differentiation and maturation of osteoblast precursors, thereby enhancing bone formation, while concurrently inhibiting osteoclastogenesis through the activation of downstream transcription factors such as LEF/TCF (82). In the context of SONFH, GCs have been shown to disrupt bone homeostasis by downregulating the expression of β-catenin and c-Myc downstream of the Wnt pathway via molecules including Dkk1, Sost, and sFRP, ultimately contributing to the pathogenesis of SONFH (83–87). Moreover, the Wnt-β-catenin pathway plays a pivotal role in bone immunoregulation, driving the polarization of synovial macrophages toward the anti-inflammatory M2 phenotype while suppressing the pro-inflammatory M1 phenotype (**Figure 1**) (88, 89).

In contrast, the non-canonical Wnt pathway exhibits greater complexity. Upon binding to Frizzled receptors, Wnt ligands can activate non-canonical signaling cascades, such as the Wnt/Ca^2+^ pathway and the Wnt/PCP pathway, which subsequently modulate the polarization of macrophages toward the M2 phenotype (90). For instance, the Wnt5a inhibits M1 macrophage polarization by suppressing the TLR4/NF-κB pathway while inducing the expression of immunosuppressive cytokines, including IL-10 and TGF-β, thereby promoting an M2-like phenotype (91). Besides, Wnt5a has been demonstrated to regulate macrophage metabolism and function through pathways such as JNK, further influencing immune responses in a context-dependent manner.

The Wnt signaling pathway serves as the central axis maintaining bone formation while suppressing bone resorption. GCs inhibit Wnt signaling, thereby impeding osteogenic differentiation and potentiating adipogenic commitment. This disruption constitutes a critical pathological basis for bone metabolic imbalance in SONFH. Inevitably, this process further exacerbates macrophage polarization imbalance, culminating in aggravated osteonecrosis.

### 2.7 MSCs promote macrophage polarization towards The M2 phenotype

During the progression of SONFH, mesenchymal stem cells (MSCs) are significantly influenced by GCs, and their regulatory effects on macrophage polarization are equally significant. MSCs suppress the activation of pro-inflammatory M1 macrophages and facilitate the anti-inflammatory M2 polarization *in vitro (* 92). MSCs facilitate the polarization of M2 macrophages via the COX-2-PGE2 signaling axis (93). BMSCs can also establish direct contact with macrophages, facilitating their polarization towards the M2 phenotype via the ICAM-1/LFA-1 interaction, which subsequently leads to an increased expression of IL-10 (94). Overall, BMSCs exert a positive effect on macrophage polarization (**Figure 1**).

The influence of macrophage polarization on osteonecrosis is unequivocally substantial, particularly in the context of GCs exposure. However, the contribution of macrophages to osteonecrosis and the regeneration of compromised osseous tissue transcends the realm of polarization. For example, OsteoMacs, a specialized subset of macrophages resident in bone, can directly differentiate into osteoclasts, thereby establishing a macrophage-osteoclast axis that is integral to bone damage precipitated by immune and inflammatory responses. The phenomenon of macrophage polarization is demonstrably prevalent across the entire temporal spectrum of osteonecrosis, exerting a considerable regulatory influence at each distinct phase of the disease’s pathogenesis (13). In the context of SONFH progression, the differentiation of macrophages into osteoclasts is frequently modulated by heightened oxidative stress, a phenomenon that encompasses the engagement of ROS. This evidence supports the hypothesis that ROS, serving as a pivotal upstream element in M1 polarization, may also be implicated in the osteoclastogenesis process (95). OsteoMacs can also respond to the bone-immune microenvironment. In response to anabolic stimuli, OsteoMacs facilitate the recruitment of BMSCs, prompting their proliferation and subsequent differentiation into osteoblastic lineage cells. Thereafter, OsteoMacs perpetuate anabolic signaling to these nascent osteoblasts. Furthermore, OsteoMacs are instrumental in the ossification process, playing a pivotal role in bone mineralization (96, 97). Therefore, while macrophage polarization plays a pivotal role in the progression of SONFH, its impact on SONFH is more diverse.

It is well-established that macrophage polarization is implicated in the pathogenesis of osteonecrosis, playing a pivotal role at each stage. However, despite recent advancements in the field of macrophage polarization research, the understanding of how these pathways and inducible factors specifically function in SONFH remains poorly understood (77, 98). In the review, we comprehensively examined the potential interactions among various immune cells within the broader context of immunity, GC, and osteonecrosis, offering novel insights that may guide research within the realm of immunology.

## 3 T cells affect the evolution of SONFH

T cell development within the thymus is a stepwise process, predominantly yielding CD4+ and CD8+ T cell subsets. Upon antigenic challenge, quiescent T cells undergo differentiation into CD4+ T helper cells and CD8+ cytotoxic effector and memory cells, which are instrumental in mediating targeted cell lysis, a spectrum of immune regulatory functions, and the establishment of long-term immunological memory (99). Empirical research has delineated that in subjects with SONFH, the proportions of CD3+ and CD4+ T lymphocytes, alongside the CD4+/CD8+ ratio, are elevated relative to healthy controls. Notably, significant disparities were exclusively identified in CD4+ lymphocyte populations. Concurrently, in SONFH rodent models, a reduction in FoxP3/CD4 double-positive regulatory T (Treg) cells within the femoral bone marrow has been observed, concomitant with an upregulation in RANKL+ T cell levels (100). Furthermore, CD4+ T lymphocytes have been implicated in enhancing RANKL and TNF levels, thereby promoting osteoclastic bone resorption and impacting both the trabecular and cortical bone compartments. In contrast, CD8+ T lymphocytes were found to upregulate TNF levels within the bone marrow, inducing a selective loss of trabecular bone (101). These findings aligned with our earlier discourse on the role of TNF and NF-κB pathways in facilitating M1 macrophage polarization. Besides, in the identification of endoplasmic reticulum stress (ERS)-related genes in SONFH, a significant association has been established between the key gene *TLR4* and CD8+ T lymphocytes (102). The above studies overlap in their assertion that T cells may play a significant role in the progression of SONFH.

**Figure 2:**
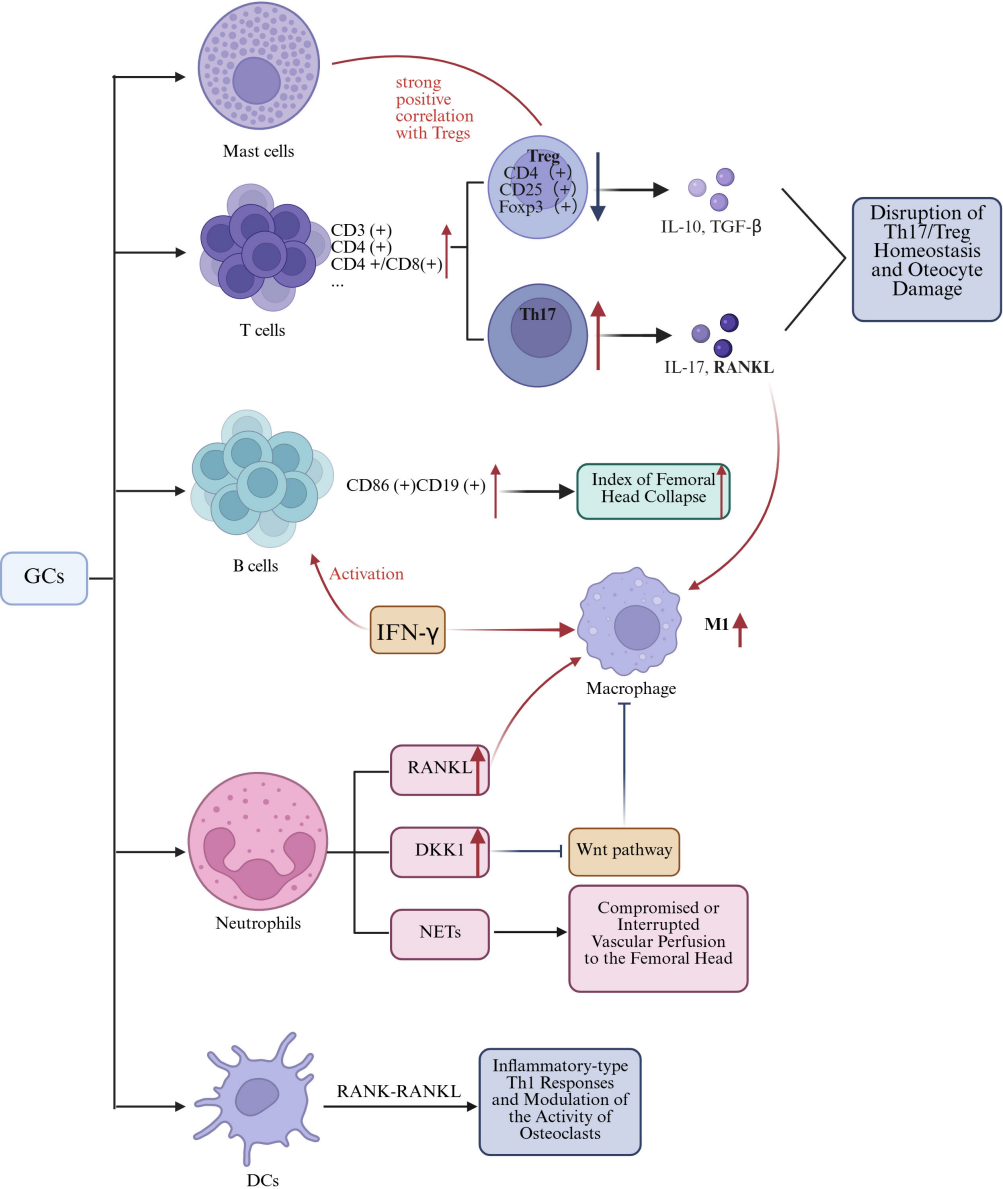
The mechanism by which GCs-induced alterations in other immune cells leads to SANFH. Figure created using BioRender..

Th17 cells represent a distinct subset of T helper cells that are capable of secreting cytokines such as interleukin-17 (IL-17). As osteoclastogenic T cells, Th17 cells play a detrimental role in bone regeneration through the production of IL-17 and RANKL (96, 103). Tregs constitute a specialized subset of T cells that possess immunosuppressive capabilities. They play a crucial role in maintaining immune tolerance and homeostasis by modulating the activity of other immune cells. In the context of bone regeneration, Tregs contribute positively to the process by creating a conducive environment for bone repair and remodeling (104). Tregs are characterized by the expression of CD4, CD25, and Foxp3, exerting their inhibitory effects on osteoclast differentiation and bone resorption through the secretion of IL-10 and TGF-β1 (105, 106). In SONFH, Tregs have been shown to correlate with the behavior of various cells, including both immune and bone cells. For instance, there is a strong positive correlation between activated mast cells and Tregs (102). Tregs can mediate the production of Wnt10b by CD8 T cells, thereby regulating bone anabolism (107). The balance between Th17 cells and Tregs has emerged as a significant area of research in immunology (108, 109). In ONFH, elevated levels of Th17 cells and interleukin-17 (IL-17) have been observed, with a positive correlation to disease severity (**Figure 2**) (110). Research indicates that the increase in Th17 cells and IL-17 is closely associated with an elevated M1/M2 macrophage ratio (111). Besides, the imbalance between Th17 cells and Tregs induces excessive IL-17 production, leading to alveolar bone damage. Modulating the ROS-macrophage polarization cascade has been shown to reshape the Th17/Treg balance, thereby promoting alveolar bone regeneration (112). Whether this mechanism can be applied to the treatment of ONFH warrants further investigation, as it may offer new therapeutic insights for this debilitating condition.

In summary, macrophage polarization imbalance constitutes the central pathogenic mechanism through which GCs disrupt the bone immune microenvironment in SONFH. However, microenvironmental homeostasis is profoundly regulated by additional immune cells. During SONFH progression, the imbalance between pro inflammatory cellular subsets including M1, Th1, and Th17 versus anti inflammatory reparative populations such as M2 and Tregs establishes a self reinforcing vicious cycle (113). This pathological cascade drives excessive inflammation that ultimately triggers osteonecrosis. The convergence of hyperinflammation, vascular injury, and enhanced osteoclast activation forms an immune network where macrophages and T cells collectively drive bone destruction and impair bone repair.

## 4 B cells promote progression of SONFH

B cells originate from hematopoietic stem cells within the bone marrow through a sequential developmental process. Initially, hematopoietic stem cells differentiate into common lymphoid progenitors, which subsequently mature into immature B cells. In contrast, immature B cells that either do not bind to self-antigens or bind with minimal affinity exit the bone marrow and, within the germinal centers, differentiate into memory B cells and plasma cells. This intricate process ensures the maturation and selection of a B cell repertoire that is both functional and self-tolerant (114). Current literature has illustrated a multifaceted relationship between B-cell activation and the process of bone healing (115). In the context of SONFH patients, Haiyu Zhang et al. revealed a significant elevation in the proportion of CD86+ CD19+ B lymphocytes compared to the healthy control cohort, with a positive correlation to the index of femoral head collapse, and a reduction of memory B cells (**Figure 2**). Moreover, a marked increase was observed in serum levels of IL-17A and IFN-γ. Notably, IFN-γ is known to activate B cells, NK cells, and macrophages, which in turn can lead to the activation of B cells and polarization towards the M1 phenotype in macrophages. Indeed, these cells are integral to the osteoclastogenesis process. Consequently, activated B cells and M1 macrophages are both involved in the bone destruction process of SONFH (116).

## 5 The role of neutrophils, DCs, and mast cells In SONFH progression

Several studies have reported a significant correlation between the percentage of neutrophils in peripheral blood and the incidence of NONFH (117, 118). Xudong Duan et al. observed a potential association between the initiation of ONFH and specific immunological parameters. Their findings suggest that there is a correlation with diminished levels of resting dendritic cells (DCs), along with heightened levels of neutrophils, monocytes, M2 macrophages, and activated dendritic cells (119). Tingyu Wu and colleagues identified a positive correlation between activated mast cells and Tregs, and observed a reduced abundance of dendritic cells in subjects with SONFH compared to the control cohort (102). Current evidence suggests that neutrophils, dendritic cells, and mast cells are directly involved in SONFH and interact through several mechanisms with various immune cells, as outlined in prior studies (**Figure 2**).

Neutrophilic polymorphonuclear leukocytes (neutrophils) are a class of terminally differentiated myeloid cells with abundant lysosomal granules, which endows them with the alternative nomenclature of granulocytes. These granules contain an extensive repertoire of potent antimicrobial armaments. Originating from hematopoietic stem cells, neutrophils represent a significant proportion of the leukocyte count in adult peripheral blood, ranging from 50% to 70% (120). Neutrophils play two critical roles: initiating acute inflammatory responses and facilitating the recruitment of monocytes and macrophages (121, 122). Research has demonstrated that neutrophils can modulate the osteogenic differentiation potential of MSCs (123). Following stimulation with LPS, neutrophils significantly upregulated RANKL expression on their membrane surface, which improved signaling interactions between neutrophils and osteoclasts, consequently enhancing the activity of osteoclast-mediated bone resorption (124). GCs induce the expression of DKK1 in leukocytes. DKK1 acts as an antagonist of the Wnt/β-catenin signaling pathway, inhibiting the propagation of Wnt signals by binding to the co-receptors LRP5/6, which are essential components of the Wnt signaling cascade (125). Current investigations into the nexus between neutrophils and orthopedic pathologies are predominantly centered on the study of neutrophil extracellular traps (NETs) (126). GCs are known to induce platelet activation, a process that not only facilitates thrombus formation, thereby precipitating ischemic alterations in the osteocytes of the femoral head, but also triggers the formation of NETs, which are hypothesized to be correlated with localized hemodynamic impairments and ischemic events within the femoral head (127, 128). Notably, the exocytosis of macrophages represents one of the pivotal mechanisms underlying the degradation of NETs. During the unique cell death process of macrophages known as METosis, structures analogous to NETs can be generated. To date, this phenomenon has been exclusively observed in the inflammatory M1 macrophage subset following stimulation with IFN-γ and LPS (129). A latest study indicated that M2-Exos exert inhibitory effects on NETs formation, consequently attenuating the pathological progression of ONFH (130). In the context of SONFH, there may exist crosstalk between macrophages and neutrophils.

Dendritic cells, which also originate from hematopoietic stem cells within the bone marrow, are extensively distributed across the body. These cells are renowned as the most potent, efficacious, and specialized antigen-presenting cells within the immune system. They are pivotal in initiating and modulating primary immune responses through the activation of naïve T cells, and are equally instrumental in the induction and perpetuation of immune tolerance under steady-state conditions (131). DCs can modulate both osteogenic differentiation and osteoclastogenesis (132, 133). In skeletal diseases, DCs may indirectly affect inflammation-related bone loss by activating and modulating the function of T cells (96). For instance, in the context of RA progression, DCs, which are typically absent from the bone marrow cavity, undergo chemotactic migration and aggregate at the site of pathology. These DCs engage in intricate interactions with T cells through the RANK-RANKL signaling axis, thereby orchestrating the activation of Th1-mediated inflammatory responses and concomitantly modulating the activity of osteoclasts to influence bone resorption processes (134). In the pathogenesis of SONFH, DCs may influence T-cell activation and differentiation. This effect can subsequently modulate the function and activity of osteoclasts, thereby contributing to the characteristic bone loss and structural deterioration observed in SONFH.

Mast cells (MCs), derived from embryonic erythro-myeloid progenitors (EMPs), are resident immune cells within tissues. Their substantial involvement in orthopedic pathologies is supported by their capacity to synthesize and store a plethora of immunomodulatory mediators that have been demonstrated to regulate bone homeostasis and contribute to skeletal disorders (135). MCs possess the capability to release cytokines associated with bone resorption, including IL-6 and RANKL (136). Furthermore, their activation has been correlated with the dysregulated chondrogenic differentiation of MSCs (137). Researchers have consistently identified a pronounced association between activated MCs and Tregs within the context of SONFH (102). These lines of evidence indicate that mast cells may likewise play a role in the pathogenesis of SONFH.

Emerging evidence suggests the critical involvement of neutrophils, DCs, and MCs in SONFH through intricate immunomodulatory networks, although their interactive mechanisms and translational potential require further elucidation. Future investigations should prioritize deciphering the multidimensional synergies among immune cells, particularly focusing on: 1) the effect of NETs in modulating local ischemia; 2) the spatiotemporal regulation of bone metabolic imbalance via dendritic cell-mediated T cell-osteoclast activation axis; and 3) the microenvironmental remodeling through MCs during bone repair. Advanced methodologies integrating single-cell multi-omics and spatial transcriptomics should be systematically employed to uncover pivotal nodes in immune-bone metabolic cross-regulation, including RANKL/OPG and Wnt/β-catenin signaling pathways, while delineating the regulatory mechanisms of glucocorticoid-induced immunometabolic reprogramming (e.g., enhanced glycolysis and succinate accumulation) on osteocyte fate determination.

## 6 Discussion

In recent years, with the use of GCs in SLE, RA, immunosuppression after organ transplantation, and other diseases, the incidence of SONFH has increased significantly. Studies have shown that the incidence of osteonecrosis is 6.7% for a cumulative dose of GCs >2 g (prednisone equivalent). For every increase of 10 mg/d, the rate of osteonecrosis increases by 3.6%, and >20 mg/d leads to a higher rate of osteonecrosis (138). The Association Research Circulation Osseous (ARCO) criteria for GA-ONFH uses GCs as the standard, requiring subjects to receive an accumulated dose of 2g or more within 3 months, with the last use of GCs within 2 years (139). As a prevalent condition with high disability rates, SONFH presents a significant clinical challenge. While THA remains the definitive intervention for end-stage disease, current conservative approaches for early-to-intermediate stages lack therapeutic efficacy, necessitating urgent exploration of novel regenerative and disease-modifying strategies in this field.

Since the formal establishment of osteoimmunology in 2000, significant progress has been made in understanding the interplay between the skeletal and immune systems (140). Nevertheless, both systems exhibit remarkable complexity: at the molecular regulatory level, the functionality of individual immune cells is governed by cross-regulatory signaling pathways with dynamic cascade interactions; at the cellular interaction level, the biological behaviors of specialized bone cells (e.g., osteoblasts and osteoclasts) are coordinately modulated by multisource immune signals, including cellular subpopulations and their secreted chemokines/cytokines. This multidimensional network of interactions suggests that traditional reductionist approaches focusing on isolated “single cell-single pathway” paradigms may lead to fragmented interpretations of disease mechanisms.

In the present review, a cascading network model of “multi-pathway regulation-macrophage polarization-bone homeostasis disruption” was established through the perspective of glucocorticoid induction. Studies have demonstrated that GCs remodel macrophage functional phenotypes via epigenetic modifications and metabolic reprogramming, while this immune cell subset acts as a regulatory hub in SONFH by secreting inflammatory mediators (e.g., IL-1β and TNF-α) and modulating the RANKL/OPG balance. This review also acknowledges certain limitations. While our focus has been on the classification of M1 and M2 macrophages, it is important to recognize that the M2 phenotype itself comprises distinct subtypes. Specifically, the M2 phenotype can be categorized into four subtypes: M2a (activated by IL-4/IL-13), M2b (induced by immune complexes, IL-1β, and LPS), M2c (activated by IL-10, TGF-β, and GCs), and M2d (TAMs, induced by IL-6, LIF, and MCSF) (14). Among these subtypes, both M2a and M2c exert anti-inflammatory effects and contribute to tissue repair, while M2b display immunoregulatory functions (141, 142). Theoretically, glucocorticoid-induced M2c macrophages possess the capacity to suppress excessive inflammatory responses, which appears beneficial for mitigating inflammatory damage in SONFH. Paradoxically, SONFH manifests complex inflammatory states and osteonecrosis. This suggests that M2c macrophages may fail to fully execute their anti-inflammatory and pro-reparative functions during this pathological process, or their actions are constrained by context-dependent constraints. Within the domain of osteoimmunology, particularly in research examining the interactions between macrophages and SONFH, our research group advocates for a focus on the dynamic regulatory mechanisms that govern the polarization of macrophage subtypes. This approach is anticipated to yield new insights for targeted therapeutic strategies aimed at mitigating the pathological progression of SONFH.

Indeed, current research in osteoimmunology, while having achieved significant advancements in understanding molecular regulatory mechanisms, still faces substantial challenges in translating these findings into clinical practice. Current therapeutic strategies primarily focus on targeted cellular interventions, such as osteoclast inhibitors, yet lack a systemic understanding of the regulatory mechanism of the osteoimmune microenvironment. Contemporary research perspectives leveraging single cell sequencing and CellChat analysis have transcended the limitations of traditional single dimensional analytical approaches (143, 144). These methodologies unlock novel investigational paradigms for osteoimmunology research. Future research should concentrate on unraveling the bidirectional crosstalk mechanisms within the immuno-osseous metabolic network, thereby establishing a theoretical framework for the development of interventional strategies aimed at reprogramming the microenvironment to restore immune homeostasis.

## References

[1] Zhao D Zhang F Wang B Liu B Li L Kim SY. Guidelines for clinical diagnosis and treatment of osteonecrosis of the femoral head in adults (2019 version). J orthopaedic translation. (2020) 21:100–10. doi: 10.1016/j.jot.2019.12.004, PMID: 32309135 PMC7152793

[2] Bernstein EM Kelsey TJ Cochran GK Deafenbaugh BK Kuhn KM. Femoral neck stress fractures: an updated review. J Am Acad Orthopaedic Surgeons. (2022) 30:302–11. doi: 10.5435/JAAOS-D-21-00398, PMID: 35077440

[3] Chona DV Minetos PD LaPrade CM Cinque ME Abrams GD Sherman SL. Hip dislocation and subluxation in athletes: A systematic review. Am J sports Med. (2022) 50:2834–41. doi: 10.1177/03635465211036104, PMID: 34623933

[4] Liao Z Jin Y Chu Y Wu H Li X Deng Z. Single-cell transcriptome analysis reveals aberrant stromal cells and heterogeneous endothelial cells in alcohol-induced osteonecrosis of the femoral head. Commun Biol. (2022) 5:324. doi: 10.1038/s42003-022-03271-6, PMID: 35388143 PMC8987047

[5] Quan H Ren C He Y Wang F Dong S Jiang H. Application of biomaterials in treating early osteonecrosis of the femoral head: Research progress and future perspectives. Acta biomaterialia. (2023) 164:15–73. doi: 10.1016/j.actbio.2023.04.005, PMID: 37080444

[6] Zhao DW Yu M Hu K Wang W Yang L Wang BJ. Prevalence of nontraumatic osteonecrosis of the femoral head and its associated risk factors in the Chinese population: results from a nationally representative survey. Chin Med J. (2015) 128:2843–50. doi: 10.4103/0366-6999.168017, PMID: 26521779 PMC4756878

[7] Horton JE Raisz LG Simmons HA Oppenheim JJ Mergenhagen SE. Bone resorbing activity in supernatant fluid from cultured human peripheral blood leukocytes. Sci (New York NY). (1972) 177:793–5. doi: 10.1126/science.177.4051.793, PMID: 5052733

[8] Yang N Liu Y. The role of the immune microenvironment in bone regeneration. Int J Med Sci. (2021) 18:3697–707. doi: 10.7150/ijms.61080, PMID: 34790042 PMC8579305

[9] Xie G Huang C Jiang S Li H Gao Y Zhang T. Smoking and osteoimmunology: Understanding the interplay between bone metabolism and immune homeostasis. J orthopaedic translation. (2024) 46:33–45. doi: 10.1016/j.jot.2024.04.003, PMID: 38765605 PMC11101877

[10] Okamoto K Nakashima T Shinohara M Negishi-Koga T Komatsu N Terashima A. Osteoimmunology: the conceptual framework unifying the immune and skeletal systems. Physiol Rev. (2017) 97:1295–349. doi: 10.1152/physrev.00036.2016, PMID: 28814613

[11] Chen K Jiao Y Liu L Huang M He C He W. Communications between bone marrow macrophages and bone cells in bone remodeling. Front Cell Dev Biol. (2020) 8:598263. doi: 10.3389/fcell.2020.598263, PMID: 33415105 PMC7783313

[12] Tan Z Wang Y Chen Y Liu Y Ma M Ma Z. The dynamic feature of macrophage M1/M2 imbalance facilitates the progression of non-traumatic osteonecrosis of the femoral head. Front Bioeng Biotechnol. (2022) 10:912133. doi: 10.3389/fbioe.2022.912133, PMID: 35573242 PMC9094367

[13] Fan S Sun X Su C Xue Y Song X Deng R. Macrophages-bone marrow mesenchymal stem cells crosstalk in bone healing. Front Cell Dev Biol. (2023) 11:1193765. doi: 10.3389/fcell.2023.1193765, PMID: 37427382 PMC10327485

[14] Abdelmagid SM Barbe MF Safadi FF. Role of inflammation in the aging bones. Life Sci. (2015) 123:25–34. doi: 10.1016/j.lfs.2014.11.011, PMID: 25510309

[15] Gao Y Xu X Zhang X. Targeting different phenotypes of macrophages: A potential strategy for natural products to treat inflammatory bone and joint diseases. Phytomedicine. (2023) 118:154952. doi: 10.1016/j.phymed.2023.154952, PMID: 37506402

[16] Mahon OR Browe DC Gonzalez-Fernandez T Pitacco P Whelan IT Von Euw S. Nano-particle mediated M2 macrophage polarization enhances bone formation and MSC osteogenesis in an IL-10 dependent manner. Biomaterials. (2020) 239:119833. doi: 10.1016/j.biomaterials.2020.119833, PMID: 32062479

[17] Ma M Tan Z Li W Zhang H Liu Y Yue C. Osteoimmunology and osteonecrosis of the femoral head. Bone Joint Res. (2022) 11:26–8. doi: 10.1302/2046-3758.111.BJR-2021-0467.R1, PMID: 35045723 PMC8801166

[18] Vi L Baht GS Whetstone H Ng A Wei Q Poon R. Macrophages promote osteoblastic differentiation *in-vivo*: implications in fracture repair and bone homeostasis. J Bone mineral Res. (2015) 30:1090–102. doi: 10.1002/jbmr.2422, PMID: 25487241

[19] Jiang C Zhou Z Lin Y Shan H Xia W Yin F. Astragaloside IV ameliorates steroid-induced osteonecrosis of the femoral head by repolarizing the phenotype of pro-inflammatory macrophages. Int Immunopharmacol. (2021) 93:107345. doi: 10.1016/j.intimp.2020.107345, PMID: 33563553

[20] Duan P Yu YL Cheng YN Nie MH Yang Q Xia LH. Exosomal miR-1a-3p derived from glucocorticoid-stimulated M1 macrophages promotes the adipogenic differentiation of BMSCs in glucocorticoid-associated osteonecrosis of the femoral head by targeting Cebpz. J Nanobiotechnol. (2024) 22:648. doi: 10.1186/s12951-024-02923-5, PMID: 39438865 PMC11494760

[21] Loi F Córdova LA Zhang R Pajarinen J Lin TH Goodman SB. The effects of immunomodulation by macrophage subsets on osteogenesis *in vitro* . Stem Cell Res Ther. (2016) 7:15. doi: 10.1186/s13287-016-0276-5, PMID: 26801095 PMC4724110

[22] Vallés G Bensiamar F Maestro-Paramio L García-Rey E Vilaboa N Saldaña L. Influence of inflammatory conditions provided by macrophages on osteogenic ability of mesenchymal stem cells. Stem Cell Res Ther. (2020) 11:57. doi: 10.1186/s13287-020-1578-1, PMID: 32054534 PMC7020593

[23] Saha S Shalova IN Biswas SK. Metabolic regulation of macrophage phenotype and function. Immunol Rev. (2017) 280:102–11. doi: 10.1111/imr.12603, PMID: 29027220

[24] Jha AK Huang SC Sergushichev A Lampropoulou V Ivanova Y Loginicheva E. Network integration of parallel metabolic and transcriptional data reveals metabolic modules that regulate macrophage polarization. Immunity. (2015) 42:419–30. doi: 10.1016/j.immuni.2015.02.005, PMID: 25786174

[25] Cargnello M Roux PP. Activation and function of the MAPKs and their substrates, the MAPK-activated protein kinases. Microbiol Mol Biol Rev. (2011) 75:50–83. doi: 10.1128/MMBR.00031-10, PMID: 21372320 PMC3063353

[26] Liu L Xiang C Li T Zhao Z Xiao T Ouyang Z. Inhibition of NF-κB and ERK signaling pathways in osteoclasts and M1 macrophage polarization: Mechanistic insights into the anti-osteoporotic effects of Pseudolaric acid B. Life Sci. (2024) 345:122592. doi: 10.1016/j.lfs.2024.122592, PMID: 38554947

[27] Wu H Dong H Tang Z Chen Y Liu Y Wang M. Electrical stimulation of piezoelectric BaTiO3 coated Ti6Al4V scaffolds promotes anti-inflammatory polarization of macrophages and bone repair via MAPK/JNK inhibition and OXPHOS activation. Biomaterials. (2023) 293:121990. doi: 10.1016/j.biomaterials.2022.121990, PMID: 36586147

[28] Lee EJ Lialios P Curtis M Williams Jt Kim Y Salipante P. Glucocorticoids alter bone microvascular barrier via MAPK/Connexin43 mechanisms. Advanced healthcare materials. (2025) 14(7):e2404302. doi: 10.1002/adhm.202404302, PMID: 39831839 PMC11912118

[29] Cotzomi-Ortega I Nieto-Yañez O Juárez-Avelar I Rojas-Sanchez G Montes-Alvarado JB Reyes-Leyva J. Autophagy inhibition in breast cancer cells induces ROS-mediated MIF expression and M1 macrophage polarization. Cell signalling. (2021) 86:110075. doi: 10.1016/j.cellsig.2021.110075, PMID: 34229086

[30] Tan C Li C Ge R Zhang W Wu Z Wang S. Mcl-1 downregulation enhances BCG treatment efficacy in bladder cancer by promoting macrophage polarization. Cancer Cell Int. (2025) 25:48. doi: 10.1186/s12935-025-03676-3, PMID: 39955585 PMC11830210

[31] Van den Bossche J Baardman J Otto NA van der Velden S Neele AE van den Berg SM. Mitochondrial dysfunction prevents repolarization of inflammatory macrophages. Cell Rep. (2016) 17:684–96. doi: 10.1016/j.celrep.2016.09.008, PMID: 27732846

[32] Stifel U Wolfschmitt EM Vogt J Wachter U Vettorazzi S Tews D. Glucocorticoids coordinate macrophage metabolism through the regulation of the tricarboxylic acid cycle. Mol Metab. (2022) 57:101424. doi: 10.1016/j.molmet.2021.101424, PMID: 34954109 PMC8783148

[33] Choi GE Han HJ. Glucocorticoid impairs mitochondrial quality control in neurons. Neurobiol disease. (2021) 152:105301. doi: 10.1016/j.nbd.2021.105301, PMID: 33609641

[34] Cheng Y Chen H Duan P Zhang H Yu Y Yu J. Early depletion of M1 macrophages retards the progression of glucocorticoid-associated osteonecrosis of the femoral head. Int Immunopharmacol. (2023) 122:110639. doi: 10.1016/j.intimp.2023.110639, PMID: 37481850

[35] Yue C Cui G Cheng Y Zhang X Sheng HF Yang Y. Aucubin suppresses TLR4/NF-κB signalling to shift macrophages toward M2 phenotype in glucocorticoid-associated osteonecrosis of the femoral head. J Cell Mol Med. (2024) 28:e18583. doi: 10.1111/jcmm.18583, PMID: 39123292 PMC11315675

[36] Liu Y Shan H Zong Y Lin Y Xia W Wang N. IKKe in osteoclast inhibits the progression of methylprednisolone-induced osteonecrosis. Int J Biol Sci. (2021) 17:1353–60. doi: 10.7150/ijbs.57962, PMID: 33867851 PMC8040464

[37] Lv B Shen N Cheng Z Chen Y Ding H Yuan J. Strategies for biomaterial-based spinal cord injury repair via the TLR4-NF-κB signaling pathway. Front Bioeng Biotechnol. (2021) 9:813169. doi: 10.3389/fbioe.2021.813169, PMID: 35600111 PMC9116428

[38] Zhang Q Sun W Li T Liu F. Polarization behavior of bone macrophage as well as associated osteoimmunity in glucocorticoid-induced osteonecrosis of the femoral head. J Inflammation Res. (2023) 16:879–94. doi: 10.2147/JIR.S401968, PMID: 36891172 PMC9986469

[39] Qin Y Hu C Jin J Chao Y Wang D Xia F. Bilobalide ameliorates osteoporosis by influencing the SIRT3/NF-κB axis in osteoclasts and promoting M2 polarization in macrophages. Int J Biol Macromol. (2024) 281:136504. doi: 10.1016/j.ijbiomac.2024.136504, PMID: 39395513

[40] Chen M Zhang Y Zhou P Liu X Zhao H Zhou X. Substrate stiffness modulates bone marrow-derived macrophage polarization through NF-κB signaling pathway. Bioact Mater. (2020) 5:880–90. doi: 10.1016/j.bioactmat.2020.05.004, PMID: 32637751 PMC7332470

[41] Wu K Yuan Y Yu H Dai X Wang S Sun Z. The gut microbial metabolite trimethylamine N-oxide aggravates GVHD by inducing M1 macrophage polarization in mice. Blood. (2020) 136:501–15. doi: 10.1182/blood.2019003990, PMID: 32291445 PMC7378459

[42] Yokota S Shimizu T Matsumae G Ebata T Alhasan H Takahashi D. Inflammasome activation in the hip synovium of rapidly destructive coxopathy patients and its relationship with the development of synovitis and bone loss. Am J Pathol. (2022) 192:794–804. doi: 10.1016/j.ajpath.2022.02.003, PMID: 35292262

[43] Fu J Wu H. Structural mechanisms of NLRP3 inflammasome assembly and activation. Annu Rev Immunol. (2023) 41:301–16. doi: 10.1146/annurev-immunol-081022-021207, PMID: 36750315 PMC10159982

[44] Murray PJ Wynn TA. Protective and pathogenic functions of macrophage subsets. Nat Rev Immunol. (2011) 11:723–37. doi: 10.1038/nri3073, PMID: 21997792 PMC3422549

[45] Chen XS Wang SH Liu CY Gao YL Meng XL Wei W. Losartan attenuates sepsis-induced cardiomyopathy by regulating macrophage polarization via TLR4-mediated NF-κB and MAPK signaling. Pharmacol Res. (2022) 185:106473. doi: 10.1016/j.phrs.2022.106473, PMID: 36182039

[46] Xue R Xie M Wu Z Wang S Zhang Y Han Z. Mesenchymal stem cell-derived exosomes promote recovery of the facial nerve injury through regulating macrophage M1 and M2 polarization by targeting the P38 MAPK/NF-Kb pathway. Aging disease. (2024) 15:851–68. doi: 10.14336/AD.2023.0719-1, PMID: 37548941 PMC10917525

[47] Toobian D Ghosh P Katkar GD. Parsing the role of PPARs in macrophage processes. Front Immunol. (2021) 12:783780. doi: 10.3389/fimmu.2021.783780, PMID: 35003101 PMC8727354

[48] Luo W Xu Q Wang Q Wu H Hua J. Effect of modulation of PPAR-γ activity on Kupffer cells M1/M2 polarization in the development of non-alcoholic fatty liver disease. Sci Rep. (2017) 7:44612. doi: 10.1038/srep44612, PMID: 28300213 PMC5353732

[49] Shao X Xu P Ji L Wu B Zhan Y Zhuang X. Low-dose decitabine promotes M2 macrophage polarization in patients with primary immune thrombocytopenia via enhancing KLF4 binding to PPARγ promoter. Clin Transl Med. (2023) 13:e1344. doi: 10.1002/ctm2.1344, PMID: 37488670 PMC10366349

[50] Luo J Wang J Zhang J Sang A Ye X Cheng Z. Nrf2 deficiency exacerbated CLP-induced pulmonary injury and inflammation through autophagy- and NF-κB/PPARγ-mediated macrophage polarization. Cells. (2022) 11:9. doi: 10.3390/cells11233927, PMID: 36497185 PMC9735993

[51] Hou J Chen J Fan J Tang Z Zhou W Lin H. Inhibition of NF-κB signaling-mediated crosstalk between macrophages and preosteoblasts by metformin alleviates trauma-induced heterotopic ossification. Inflammation. (2023) 46:1414–29. doi: 10.1007/s10753-023-01817-2, PMID: 37115368

[52] Liu F Zhao Y Pei Y Lian F Lin H. Role of the NF-kB signalling pathway in heterotopic ossification: biological and therapeutic significance. Cell Commun Signal. (2024) 22:159. doi: 10.1186/s12964-024-01533-w, PMID: 38439078 PMC10910758

[53] Fu J Zhang J Jiang T Ao X Li P Lian Z. mTORC1 coordinates NF-κB signaling pathway to promote chondrogenic differentiation of tendon cells in heterotopic ossification. Bone. (2022) 163:116507. doi: 10.1016/j.bone.2022.116507, PMID: 35908648

[54] Cristancho AG Lazar MA. Forming functional fat: a growing understanding of adipocyte differentiation. Nat Rev Mol Cell Biol. (2011) 12:722–34. doi: 10.1038/nrm3198, PMID: 21952300 PMC7171550

[55] Han L Wang B Wang R Gong S Chen G Xu W. The shift in the balance between osteoblastogenesis and adipogenesis of mesenchymal stem cells mediated by glucocorticoid receptor. Stem Cell Res Ther. (2019) 10:377. doi: 10.1186/s13287-019-1498-0, PMID: 31805987 PMC6896503

[56] Heming M Gran S Jauch SL Fischer-Riepe L Russo A Klotz L. Peroxisome proliferator-activated receptor-γ Modulates the response of macrophages to lipopolysaccharide and glucocorticoids. Front Immunol. (2018) 9:893. doi: 10.3389/fimmu.2018.00893, PMID: 29867927 PMC5949563

[57] Tu Y Liu J Kong D Guo X Li J Long Z. Irisin drives macrophage anti-inflammatory differentiation via JAK2-STAT6-dependent activation of PPARγ and Nrf2 signaling. Free Radic Biol Med. (2023) 201:98–110. doi: 10.1016/j.freeradbiomed.2023.03.014, PMID: 36940733

[58] Isali I McClellan P Shankar E Gupta S Jain M Anderson JM. Genipin guides and sustains the polarization of macrophages to the pro-regenerative M2 subtype via activation of the pSTAT6-PPAR-gamma pathway. Acta biomaterialia. (2021) 131:198–210. doi: 10.1016/j.actbio.2021.06.043, PMID: 34224892 PMC8373816

[59] Fang S Wu S Chen P. Alpha-2-macroglobulin mitigates glucocorticoid-induced osteonecrosis via Keap1/Nrf2 pathway activation. Free Radic Biol Med. (2024) 225:501–16. doi: 10.1016/j.freeradbiomed.2024.09.048, PMID: 39343183

[60] He L Jhong JH Chen Q Huang KY Strittmatter K Kreuzer J. Global characterization of macrophage polarization mechanisms and identification of M2-type polarization inhibitors. Cell Rep. (2021) 37:109955. doi: 10.1016/j.celrep.2021.109955, PMID: 34731634 PMC8783961

[61] Panwar V Singh A Bhatt M Tonk RK Azizov S Raza AS. Multifaceted role of mTOR (mammalian target of rapamycin) signaling pathway in human health and disease. Signal Transduct Target Ther. (2023) 8:375. doi: 10.1038/s41392-023-01608-z, PMID: 37779156 PMC10543444

[62] Wu MM Wang QM Huang BY Mai CT Wang CL Wang TT. Dioscin ameliorates murine ulcerative colitis by regulating macrophage polarization. Pharmacol Res. (2021) 172:105796. doi: 10.1016/j.phrs.2021.105796, PMID: 34343656

[63] Zhang X Chen C Ling C Luo S Xiong Z Liu X. EGFR tyrosine kinase activity and Rab GTPases coordinate EGFR trafficking to regulate macrophage activation in sepsis. Cell Death Dis. (2022) 13:934. doi: 10.1038/s41419-022-05370-y, PMID: 36344490 PMC9640671

[64] Cui Y Chen J Zhang Z Shi H Sun W Yi Q. The role of AMPK in macrophage metabolism, function and polarisation. J Transl Med. (2023) 21:892. doi: 10.1186/s12967-023-04772-6, PMID: 38066566 PMC10709986

[65] Ye W Wang J Lin D Ding Z. The immunomodulatory role of irisin on osteogenesis via AMPK-mediated macrophage polarization. Int J Biol Macromol. (2020) 146:25–35. doi: 10.1016/j.ijbiomac.2019.12.028, PMID: 31843619

[66] Zhu YP Brown JR Sag D Zhang L Suttles J. Adenosine 5’-monophosphate-activated protein kinase regulates IL-10-mediated anti-inflammatory signaling pathways in macrophages. J Immunol. (2015) 194:584–94. doi: 10.4049/jimmunol.1401024, PMID: 25512602 PMC4343033

[67] Caratti G Desgeorges T Juban G Stifel U Fessard A Koenen M. Macrophagic AMPKα1 orchestrates regenerative inflammation induced by glucocorticoids. EMBO Rep. (2023) 24:e55363. doi: 10.15252/embr.202255363, PMID: 36520372 PMC9900347

[68] Tang X Zhu M Zhu Z Tang W Zhang H Chen Y. Ginsenoside Re inhibits non-small cell lung cancer progression by suppressing macrophage M2 polarization induced by AMPKα1/STING positive feedback loop. Phytother research: PTR. (2024) 38:5088–106. doi: 10.1002/ptr.8309, PMID: 39119862

[69] Shuid AN Abdul Nasir NA Ab Azis N Shuid AN Razali N Ahmad Hairi H. A systematic review on the molecular mechanisms of resveratrol in protecting against osteoporosis. Int J Mol Sci. (2025) 26:15. doi: 10.3390/ijms26072893, PMID: 40243497 PMC11988631

[70] Zhang Y Cui Y Sun C Guo J Li M. ED-71 ameliorates OVX-induced osteoporosis by regulating calcium homeostasis and SIRT1-mediated mitochondrial function, alleviating osteoblast senescence and suppressing osteoclastogenesis. Cell signalling. (2025) 131:111713. doi: 10.1016/j.cellsig.2025.111713, PMID: 40049265

[71] Chen L Wang BZ Xie J Zhang RY Jin C Chen WK. Therapeutic effect of SIRT3 on glucocorticoid-induced osteonecrosis of the femoral head via intracellular oxidative suppression. Free Radic Biol Med. (2021) 176:228–40. doi: 10.1016/j.freeradbiomed.2021.07.016, PMID: 34260898

[72] Shen Y Jiang B Lu W Luo B Zhou Y Qian G. Dexamethasone-induced mitochondrial ROS-mediated inhibition of AMPK activity facilitates osteoblast necroptosis. Toxicol Res. (2023) 12:922–9. doi: 10.1093/toxres/tfad080, PMID: 37915480 PMC10615823

[73] Zhao SJ Kong FQ Jie J Li Q Liu H Xu AD. Macrophage MSR1 promotes BMSC osteogenic differentiation and M2-like polarization by activating PI3K/AKT/GSK3β/β-catenin pathway. Theranostics. (2020) 10:17–35. doi: 10.7150/thno.36930, PMID: 31903103 PMC6929615

[74] Fu L Wu W Sun X Zhang P. Glucocorticoids enhanced osteoclast autophagy through the PI3K/Akt/mTOR signaling pathway. Calcified Tissue Int. (2020) 107:60–71. doi: 10.1007/s00223-020-00687-2, PMID: 32274533

[75] Lin Y Chen M Guo W Qiu S Chen L Liu W. Zoledronic acid relieves steroid-induced avascular necrosis of femoral head via inhibiting FOXD3 mediated ANXA2 transcriptional activation. Bone. (2024) 188:117222. doi: 10.1016/j.bone.2024.117222, PMID: 39102974

[76] Ma J Shen M Yue D Wang W Gao F Wang B. Extracellular vesicles from BMSCs prevent glucocorticoid-induced BMECs injury by regulating autophagy via the PI3K/Akt/mTOR pathway. Cells. (2022) 11:11. doi: 10.3390/cells11132104, PMID: 35805188 PMC9265732

[77] Fernandes TL Gomoll AH Lattermann C Hernandez AJ Bueno DF Amano MT. Macrophage: A potential target on cartilage regeneration. Front Immunol. (2020) 11:111. doi: 10.3389/fimmu.2020.00111, PMID: 32117263 PMC7026000

[78] Zhu R Yang G Cao Z Shen K Zheng L Xiao J. The prospect of serum and glucocorticoid-inducible kinase 1 (SGK1) in cancer therapy: a rising star. Ther Adv Med Oncol. (2020) 12:1758835920940946. doi: 10.1177/1758835920940946, PMID: 32728395 PMC7364809

[79] Wu Y Zhan S Chen L Sun M Li M Mou X. TNFSF14/LIGHT promotes cardiac fibrosis and atrial fibrillation vulnerability via PI3Kγ/SGK1 pathway-dependent M2 macrophage polarisation. J Transl Med. (2023) 21:544. doi: 10.1186/s12967-023-04381-3, PMID: 37580750 PMC10424430

[80] Han X Wang F Ma Y Lv X Zhang K Wang Y. TPG-functionalized PLGA/PCL nanofiber membrane facilitates periodontal tissue regeneration by modulating macrophages polarization via suppressing PI3K/AKT and NF-κB signaling pathways. Mater Today Bio. (2024) 26:101036. doi: 10.1016/j.mtbio.2024.101036, PMID: 38600919 PMC11004206

[81] Chae WJ Bothwell ALM. Canonical and non-canonical Wnt signaling in immune cells. Trends Immunol. (2018) 39:830–47. doi: 10.1016/j.it.2018.08.006, PMID: 30213499 PMC7367500

[82] Xia C Xu H Fang L Chen J Yuan W Fu D. β-catenin inhibition disrupts the homeostasis of osteogenic/adipogenic differentiation leading to the development of glucocorticoid-induced osteonecrosis of the femoral head. eLife. (2024) 12:2. doi: 10.7554/eLife.92469, PMID: 38376133 PMC10942600

[83] Meszaros K Patocs A. Glucocorticoids influencing Wnt/β-catenin pathway; multiple sites, heterogeneous effects. Molecules (Basel Switzerland). (2020) 25:3–4. doi: 10.3390/molecules25071489, PMID: 32218328 PMC7181001

[84] Wu J Chen T Zhang M Li X Fu R Xu J. Atorvastatin exerts a preventive effect against steroid-induced necrosis of the femoral head by modulating Wnt5a release. Arch toxicol. (2024) 98:3365–80. doi: 10.1007/s00204-024-03817-z, PMID: 38971901

[85] Chen XJ Shen YS He MC Yang F Yang P Pang FX. Polydatin promotes the osteogenic differentiation of human bone mesenchymal stem cells by activating the BMP2-Wnt/β-catenin signaling pathway. Biomedicine pharmacotherapy = Biomedecine pharmacotherapie. (2019) 112:108746. doi: 10.1016/j.biopha.2019.108746, PMID: 30970530

[86] Zhang C Zou YL Ma J Dang XQ Wang KZ. Apoptosis associated with Wnt/β-catenin pathway leads to steroid-induced avascular necrosis of femoral head. BMC musculoskeletal Disord. (2015) 16:132. doi: 10.1186/s12891-015-0606-2, PMID: 26037065 PMC4453221

[87] Wang LT Chen LR Chen KH. Hormone-related and drug-induced osteoporosis: A cellular and molecular overview. Int J Mol Sci. (2023) 24:10. doi: 10.3390/ijms24065814, PMID: 36982891 PMC10054048

[88] Liu Y Hao R Lv J Yuan J Wang X Xu C. Targeted knockdown of PGAM5 in synovial macrophages efficiently alleviates osteoarthritis. Bone Res. (2024) 12:15. doi: 10.1038/s41413-024-00318-8, PMID: 38433252 PMC10909856

[89] Liu Y Lv H Liu X Xu L Li T Zhou H. The RP11-417E7.1/THBS2 signaling pathway promotes colorectal cancer metastasis by activating the Wnt/β-catenin pathway and facilitating exosome-mediated M2 macrophage polarization. J Exp Clin Cancer research: CR. (2024) 43:195. doi: 10.1186/s13046-024-03107-7, PMID: 39020380 PMC11253389

[90] Abaricia JO Shah AH Chaubal M Hotchkiss KM Olivares-Navarrete R. Wnt signaling modulates macrophage polarization and is regulated by biomaterial surface properties. Biomaterials. (2020) 243:119920. doi: 10.1016/j.biomaterials.2020.119920, PMID: 32179303 PMC7191325

[91] Bergenfelz C Medrek C Ekström E Jirström K Janols H Wullt M. Wnt5a induces a tolerogenic phenotype of macrophages in sepsis and breast cancer patients. J Immunol. (2012) 188:5448–58. doi: 10.4049/jimmunol.1103378, PMID: 22547701

[92] Harrell CR Markovic BS Fellabaum C Arsenijevic A Volarevic V. Mesenchymal stem cell-based therapy of osteoarthritis: Current knowledge and future perspectives. Biomedicine pharmacotherapy = Biomedecine pharmacotherapie. (2019) 109:2318–26. doi: 10.1016/j.biopha.2018.11.099, PMID: 30551490

[93] Jin L Deng Z Zhang J Yang C Liu J Han W. Mesenchymal stem cells promote type 2 macrophage polarization to ameliorate the myocardial injury caused by diabetic cardiomyopathy. J Transl Med. (2019) 17:251. doi: 10.1186/s12967-019-1999-8, PMID: 31382970 PMC6683374

[94] Takizawa N Okubo N Kamo M Chosa N Mikami T Suzuki K. Bone marrow-derived mesenchymal stem cells propagate immunosuppressive/anti-inflammatory macrophages in cell-to-cell contact-independent and -dependent manners under hypoxic culture. Exp Cell Res. (2017) 358:411–20. doi: 10.1016/j.yexcr.2017.07.014, PMID: 28712928

[95] Zhang X Feng C Yuan T Wang Y Wang H Lu Q. Inhibition of protein disulfide isomerase mitigates steroid-induced osteonecrosis of the femoral head by suppressing osteoclast activity through the reduction of cellular oxidative stress. Chem Biol Interact. (2024) 404:111263. doi: 10.1016/j.cbi.2024.111263, PMID: 39393751

[96] Miron RJ Bohner M Zhang Y Bosshardt DD. Osteoinduction and osteoimmunology: Emerging concepts. Periodontol 2000. (2024) 94:9–26. doi: 10.1111/prd.12519, PMID: 37658591

[97] Sun Y Li J Xie X Gu F Sui Z Zhang K. Macrophage-osteoclast associations: origin, polarization, and subgroups. Front Immunol. (2021) 12:778078. doi: 10.3389/fimmu.2021.778078, PMID: 34925351 PMC8672114

[98] Chen S Saeed A Liu Q Jiang Q Xu H Xiao GG. Macrophages in immunoregulation and therapeutics. Signal Transduct Target Ther. (2023) 8:207. doi: 10.1038/s41392-023-01452-1, PMID: 37211559 PMC10200802

[99] Sun L Su Y Jiao A Wang X Zhang B. T cells in health and disease. Signal Transduct Target Ther. (2023) 8:235. doi: 10.1038/s41392-023-01471-y, PMID: 37332039 PMC10277291

[100] Chen C Zhao X Luo Y Li B Li Q Zhao C. Imbalanced T-cell subsets may facilitate the occurrence of osteonecrosis of the femoral head. J Inflammation Res. (2022) 15:4159–69. doi: 10.2147/JIR.S367214, PMID: 35912401 PMC9328079

[101] Weitzmann MN Vikulina T Roser-Page S Yamaguchi M Ofotokun I. Homeostatic expansion of CD4+ T cells promotes cortical and trabecular bone loss, whereas CD8+ T cells induce trabecular bone loss only. J Infect diseases. (2017) 216:1070–9. doi: 10.1093/infdis/jix444, PMID: 28968828 PMC5853557

[102] Wu T Shi W Zhou Y Guo S Tian H Jiang Y. Identification and validation of endoplasmic reticulum stress-related genes in patients with steroid-induced osteonecrosis of the femoral head. Sci Rep. (2024) 14:21634. doi: 10.1038/s41598-024-72941-8, PMID: 39284931 PMC11405670

[103] Sato K Suematsu A Okamoto K Yamaguchi A Morishita Y Kadono Y. Th17 functions as an osteoclastogenic helper T cell subset that links T cell activation and bone destruction. J Exp Med. (2006) 203:2673–82. doi: 10.1084/jem.20061775, PMID: 17088434 PMC2118166

[104] Griffith JW Luster AD. No bones about it: regulatory T cells promote fracture healing. J Clin Invest. (2025) 135:1-2. doi: 10.1172/JCI188368, PMID: 39817452 PMC11735088

[105] Luo CY Wang L Sun C Li DJ. Estrogen enhances the functions of CD4(+)CD25(+)Foxp3(+) regulatory T cells that suppress osteoclast differentiation and bone resorption *in vitro* . Cell Mol Immunol. (2011) 8:50–8. doi: 10.1038/cmi.2010.54, PMID: 21200384 PMC4002989

[106] Zaiss MM Frey B Hess A Zwerina J Luther J Nimmerjahn F. Regulatory T cells protect from local and systemic bone destruction in arthritis. J Immunol. (2010) 184:7238–46. doi: 10.4049/jimmunol.0903841, PMID: 20483756

[107] Tyagi AM Yu M Darby TM Vaccaro C Li JY Owens JA. The microbial metabolite butyrate stimulates bone formation via T regulatory cell-mediated regulation of WNT10B expression. Immunity. (2018) 49:1116–31.e7. doi: 10.1016/j.immuni.2018.10.013, PMID: 30446387 PMC6345170

[108] Knochelmann HM Dwyer CJ Bailey SR Amaya SM Elston DM Mazza-McCrann JM. When worlds collide: Th17 and Treg cells in cancer and autoimmunity. Cell Mol Immunol. (2018) 15:458–69. doi: 10.1038/s41423-018-0004-4, PMID: 29563615 PMC6068176

[109] Kikuiri T Kim I Yamaza T Akiyama K Zhang Q Li Y. Cell-based immunotherapy with mesenchymal stem cells cures bisphosphonate-related osteonecrosis of the jaw-like disease in mice. J Bone mineral Res. (2010) 25:1668–79. doi: 10.1002/jbmr.37, PMID: 20200952 PMC3154005

[110] Zou D Zhang K Yang Y Ren Y Zhang L Xiao X. Th17 and IL-17 exhibit higher levels in osteonecrosis of the femoral head and have a positive correlation with severity of pain. Endokrynologia Polska. (2018) 69:283–90. doi: 10.5603/EP.a2018.0031, PMID: 29952419

[111] Zhang Q Atsuta I Liu S Chen C Shi S Shi S. IL-17-mediated M1/M2 macrophage alteration contributes to pathogenesis of bisphosphonate-related osteonecrosis of the jaws. Clin Cancer Res. (2013) 19:3176–88. doi: 10.1158/1078-0432.CCR-13-0042, PMID: 23616636 PMC5558149

[112] Li J Li M Zhang C Fei Y Wang Y Zhong Z. Active targeting microemulsion-based thermosensitive hydrogel against periodontitis by reconstructing Th17/Treg homeostasis via regulating ROS-macrophages polarization cascade. Int J Pharm. (2024) 659:124263. doi: 10.1016/j.ijpharm.2024.124263, PMID: 38815639

[113] Tu J Huang W Zhang W Mei J Zhu C. A tale of two immune cells in rheumatoid arthritis: the crosstalk between macrophages and T cells in the synovium. Front Immunol. (2021) 12:655477. doi: 10.3389/fimmu.2021.655477, PMID: 34220809 PMC8248486

[114] Tangye SG Nguyen T Deenick EK Bryant VL Ma CS. Inborn errors of human B cell development, differentiation, and function. J Exp Med. (2023) 220:12. doi: 10.1084/jem.20221105, PMID: 37273190 PMC10242086

[115] Yang HZ Zhan Y Liu Y Guo M Fan Y Luo G. NIR-stimulated rGO-HAMC hydrogel enhances fracture healing through regulating B-cell signaling. Biomaterials advances. (2025) 166:214080. doi: 10.1016/j.bioadv.2024.214080, PMID: 39490190

[116] Zhang H Xiao F Liu Y Zhao D Shan Y Jiang Y. A higher frequency of peripheral blood activated B cells in patients with non-traumatic osteonecrosis of the femoral head. Int Immunopharmacol. (2014) 20:95–100. doi: 10.1016/j.intimp.2014.02.016, PMID: 24583150

[117] Jiang J Liu X Lai B Hu D Lai L Xu J. Correlational analysis between neutrophil granulocyte levels and osteonecrosis of the femoral head. BMC musculoskeletal Disord. (2019) 20:393. doi: 10.1186/s12891-019-2778-7, PMID: 31470845 PMC6717348

[118] Wang Y Li D Chen H Li Z Feng B Weng X. Accumulation of fat not responsible for femoral head necrosis, revealed by single-cell RNA sequencing: A preliminary study. Biomolecules. (2023) 13:18. doi: 10.3390/biom13010171, PMID: 36671556 PMC9856115

[119] Duan X Xing F Zhang J Li H Chen Y Lei Y. Bioinformatic analysis of related immune cell infiltration and key genes in the progression of osteonecrosis of the femoral head. Front Immunol. (2024) 14:1340446. doi: 10.3389/fimmu.2023.1340446, PMID: 38283345 PMC10811953

[120] Chapple ILC Hirschfeld J Kantarci A Wilensky A Shapira L. The role of the host-Neutrophil biology. Periodontol 2000. (2023) 1. doi: 10.1111/prd.12490, PMID: 37199393

[121] Kolaczkowska E Kubes P. Neutrophil recruitment and function in health and inflammation. Nat Rev Immunol. (2013) 13:159–75. doi: 10.1038/nri3399, PMID: 23435331

[122] Kovtun A Bergdolt S Wiegner R Radermacher P Huber-Lang M Ignatius A. The crucial role of neutrophil granulocytes in bone fracture healing. Eur Cells materials. (2016) 32:152–62. doi: 10.22203/eCM.v032a10, PMID: 27452963

[123] Feng X Wang C Ji B Qiao J Xu Y Zhu S. CD_99 G1 neutrophils modulate osteogenic differentiation of mesenchymal stem cells in the pathological process of ankylosing spondylitis. Ann rheumatic diseases. (2024) 83:324–34. doi: 10.1136/ard-2023-224107, PMID: 37977819 PMC10894850

[124] Chakravarti A Raquil MA Tessier P Poubelle PE. Surface RANKL of Toll-like receptor 4-stimulated human neutrophils activates osteoclastic bone resorption. Blood. (2009) 114:1633–44. doi: 10.1182/blood-2008-09-178301, PMID: 19546479

[125] Brunetti G Faienza MF Piacente L Ventura A Oranger A Carbone C. High dickkopf-1 levels in sera and leukocytes from children with 21-hydroxylase deficiency on chronic glucocorticoid treatment. Am J Physiol Endocrinol Metab. (2013) 304:E546–54. doi: 10.1152/ajpendo.00535.2012, PMID: 23299503

[126] Xiang M Yin M Xie S Shi L Nie W Shi B. The molecular mechanism of neutrophil extracellular traps and its role in bone and joint disease. Heliyon. (2023) 9:e22920. doi: 10.1016/j.heliyon.2023.e22920, PMID: 38076128 PMC10703630

[127] Nonokawa M Shimizu T Yoshinari M Hashimoto Y Nakamura Y Takahashi D. Association of neutrophil extracellular traps with the development of idiopathic osteonecrosis of the femoral head. Am J Pathol. (2020) 190:2282–9. doi: 10.1016/j.ajpath.2020.07.008, PMID: 32702358

[128] Ogawa H Yokota S Hosoi Y Shindo A Ogawa N Yamamura R. Methylprednisolone pulse-enhanced neutrophil extracellular trap formation in mice with imiquimod-induced lupus-like disease, resulting in ischaemia of the femoral head cartilage. Lupus Sci Med. (2023) 10:4. doi: 10.1136/lupus-2023-001042, PMID: 38154828 PMC10759060

[129] Fang Q Stehr AM Naschberger E Knopf J Herrmann M Stürzl M. No NETs no TIME: Crosstalk between neutrophil extracellular traps and the tumor immune microenvironment. Front Immunol. (2022) 13:1075260. doi: 10.3389/fimmu.2022.1075260, PMID: 36618417 PMC9816414

[130] Liu G Cao R Liu Q Li H Yan P Wang K. M2 macrophages-derived exosomes for osteonecrosis of femoral head treatment: modulating neutrophil extracellular traps formation and endothelial phenotype transition. Bone Res. (2025) 13:42. doi: 10.1038/s41413-025-00412-5, PMID: 40169566 PMC11961764

[131] Zanna MY Yasmin AR Omar AR Arshad SS Mariatulqabtiah AR Nur-Fazila SH. Review of dendritic cells, their role in clinical immunology, and distribution in various animal species. Int J Mol Sci. (2021) 22:7,20. doi: 10.3390/ijms22158044, PMID: 34360810 PMC8348663

[132] Cao Z Wu Y Yu L Zou L Yang L Lin S. Exosomal miR-335 derived from mature dendritic cells enhanced mesenchymal stem cell-mediated bone regeneration of bone defects in athymic rats. Mol Med (Cambridge Mass). (2021) 27:20. doi: 10.1186/s10020-021-00268-5, PMID: 33637046 PMC7913386

[133] Quan H Ren C Xie H He Z Ding H Li J. An injectable hydrogel loaded with miRNA nanocarriers promotes vessel-associated osteoclast (VAO)-mediated angiogenesis and bone regeneration in osteonecrosis of the rat femoral head. Biomaterials. (2025) 320:123252. doi: 10.1016/j.biomaterials.2025.123252, PMID: 40081225

[134] Santiago-Schwarz F Anand P Liu S Carsons SE. Dendritic cells (DCs) in rheumatoid arthritis (RA): progenitor cells and soluble factors contained in RA synovial fluid yield a subset of myeloid DCs that preferentially activate Th1 inflammatory-type responses. J Immunol. (2001) 167:1758–68. doi: 10.4049/jimmunol.167.3.1758, PMID: 11466401

[135] Ragipoglu D Dudeck A Haffner-Luntzer M Voss M Kroner J Ignatius A. The role of mast cells in bone metabolism and bone disorders. Front Immunol. (2020) 11:163. doi: 10.3389/fimmu.2020.00163, PMID: 32117297 PMC7025484

[136] Fischer V Haffner-Luntzer M. Interaction between bone and immune cells: Implications for postmenopausal osteoporosis. Semin Cell Dev Biol. (2022) 123:14–21. doi: 10.1016/j.semcdb.2021.05.014, PMID: 34024716

[137] Jiang T Ao X Xiang X Zhang J Cai J Fu J. Mast cell activation by NGF drives the formation of trauma-induced heterotopic ossification. JCI Insight. (2024) 10:13–6. doi: 10.1172/jci.insight.179759, PMID: 39589893 PMC11721298

[138] Mont MA Pivec R Banerjee S Issa K Elmallah RK Jones LC. High-dose corticosteroid use and risk of hip osteonecrosis: meta-analysis and systematic literature review. J arthroplasty. (2015) 30:1506–12.e5. doi: 10.1016/j.arth.2015.03.036, PMID: 25900167 PMC7127809

[139] Yoon BH Jones LC Chen CH Cheng EY Cui Q Drescher W. Etiologic classification criteria of ARCO on femoral head osteonecrosis part 1: glucocorticoid-associated osteonecrosis. J arthroplasty. (2019) 34:163–8.e1. doi: 10.1016/j.arth.2018.09.005, PMID: 30348552

[140] Arron JR Choi Y. Bone versus immune system. Nature. (2000) 408:535–6. doi: 10.1038/35046196, PMID: 11117729

[141] Zhao L Tang S Chen F Ren X Han X Zhou X. Regulation of macrophage polarization by targeted metabolic reprogramming for the treatment of lupus nephritis. Mol Med (Cambridge Mass). (2024) 30:96. doi: 10.1186/s10020-024-00866-z, PMID: 38914953 PMC11197188

[142] Hou Y Wei D Zhang Z Lei T Li S Bao J. Downregulation of nutrition sensor GCN2 in macrophages contributes to poor wound healing in diabetes. Cell Rep. (2024) 43:113658. doi: 10.1016/j.celrep.2023.113658, PMID: 38175755

[143] Su N Saqib H Villicana C Kim S Ambrosi TH Freeman P. Modulating immune-stem cell crosstalk enables robust bone regeneration via tuning compositions of macroporous scaffolds. NPJ Regenerative Med. (2025) 10:33. doi: 10.1038/s41536-025-00421-2, PMID: 40595820 PMC12219738

[144] Zhou X Zhang C Yang S Yang L Luo W Zhang W. Macrophage-derived MMP12 promotes fibrosis through sustained damage to endothelial cells. J hazardous materials. (2024) 461:132733. doi: 10.1016/j.jhazmat.2023.132733, PMID: 37816293

